# New York Quarantine Establishment

**Published:** 1858-09

**Authors:** 


NEW YORK QUARANTINE ESTABLISHMENT.

The Quarantine buildings on Staten Island, connected with the port of New York, were deliberately burned to the ground, on the nights of the 1st and 2d of September, by the inhabitants of the neighborhood, acting in the capacity of a mob. The patients, amounting to a large number, were first taken out into the open air and left without a shelter. Fear of the spread of yellow fever was the alleged cause; but such an act of vandalism is a disgrace to our age and nation, which admits of neither palliation nor excuse.

